# End-user frustrations and failures in digital technology: exploring the role of Fear of Missing Out, Internet addiction and personality

**DOI:** 10.1016/j.heliyon.2018.e00872

**Published:** 2018-11-01

**Authors:** Lee Hadlington, Mark O. Scase

**Affiliations:** De Montfort University, The Gateway, Leicester, LE1 9BH, UK

**Keywords:** Psychology

## Abstract

The present study aimed to explore the potential relationship between individual differences in responses to failures with digital technology. In total, 630 participants (50% male) aged between 18–68 years (*M* = 41.41, *SD* = 14.18) completed an online questionnaire. This included a self-report, response to failures in digital technology scale, a measure of Fear of Missing Out, Internet addiction, and the BIG-5 personality traits. Fear of Missing Out, Internet addiction, extraversion, and neuroticism all served as significant positive predictors for maladaptive responses to failures in digital technology. Agreeableness, conscientiousness, and openness acted as significant negative predictors for maladaptive responses to failures in digital technology. The responses to failures in digital technology scale presented good internal reliability, with items loading onto four key factors, these being; ‘maladaptive responses’, ‘adaptive responses’, ‘external support and venting frustrations’, and ‘anger and resignation’. The findings are discussed in the context of the end user experience, particularly where individual differences are seen to influence the level of frustration arising from a failure. The findings are also seen as a potential route for reducing the negative impact of failures in digital technology, particularly in the context of organisational productivity and responses to malicious cyberattacks.

## 1 Introduction

In the context of Human-Computer Interaction (HCI), there is general agreement that emotion plays a key role in the user experience (Buck et al., 2017; Jokinen, 2015; Saariluomaand and Jokinen, 2014). In fact, Jokinen (2015) noted that the term ‘user experience’ had been widely adopted in the field of HCI to reflect a focus on the feelings end users experience when interacting with digital technology. Jokinen (2015) suggested that successful HCI experiences are viewed in a positive way, especially when they are congruent with the goals of the end user. However there are instances where frustration, anxiety, and confusion can arise, resulting in experiences that are incongruent with the goals of the end user (Jokinen, 2015). One potential source for frustration to arise is in situations where digital technology responds in a way that is not conducive to the goals of the end user. Such failures in digital technology have been noted to be commonplace, with estimates suggested that experiences resulting in some form of frustrating response account for between 30.5–45.9% of the time an individual spends on a computer (Ceaparu et al., 2004). Jokinen (2015) noted that although frustration was a key element of the emotional user experience, not all individuals react in this way when encountering issues with digital technology. Individual differences serve to moderate the way in which an individual deals with a potential frustration-eliciting event, meaning that some may implement alternative actions to circumvent such issues (Jokinen, 2015).

To date, there exists no comprehensive examination of the relationship between individual differences and the level of frustration experienced in the context of failures in digital technology. In practical terms, gaining a more detailed understanding of how individuals react to failures in digital technology could lead to the development of interventions, limiting negative and maladaptive responses to digital technology failures. This also has the potential to build a level of resilience in the end user experience, particularly given the growing potential for widespread disruption from malicious cyberattacks (Gross et al., 2016, 2017). From a theoretical perspective, it is envisaged that this work could add to the existing work already being conducted exploring the role of emotion in the end user experience.

### 1.1 Conceptualising frustration as a response

There has been a well-documented discussion about the nature and definition of frustration (e.g. Berkowitz, 1989). Frustration can be viewed both as an external event that has an impact on the individual, or the emotive response that the individual experiences (Berkowitz, 1989; Britt and Janus, 1940). Lazar et al. (2006a) suggested that frustrations occur ‘*when there is an inhibiting condition that interferes with or stops the realization of a goal*’ (p. 189). In the context of the present study, frustration is viewed as the emotive response to an external event, more specifically a failure associated with digital technology, rather than the external event itself. This approach adopted in the current paper builds upon previous research that proposed a taxonomy of frustration, with particular focus on the emotive elements (Britt and Janus, 1940; Lazar et al., 2006a; Rosenzweig, 1934; Shorkey and Crocker, 1981).

Frustration can be viewed in terms of being either adaptive or maladaptive in nature (Britt and Janus, 1940; Rosenzweig, 1934; Shorkey and Crocker, 1981). Such adaptive and maladaptive responses to external stressors have also been widely discussed in the context of health psychology (e.g. Bonne et al., 2004; Borsook et al., 2012). Adaptive responses include using problem solving strategies to circumvent the problem once it is encountered. Two forms of adaptive responses have been identified in the previous literature, these being (a) transforming stress into active energy and reapplying this to the current goal, or (b) the identification and pursuit of alternative goals (Britt and Janus, 1940; Shorkey and Crocker, 1981).

Maladaptive responses are typified by a lack of constructive problem solving that ultimately leads to the creation of additional problems. Britt and Janus (1940) suggested that these responses can be classified as being either objective responses (outwardly observable) or subjective (conscious responses to the frustrating event). Objective responses can include aspects such as aggression, withdrawal, regression, fixation, and resignation. For example, an individual frustration could be displayed in an overtly aggressive manner. Previous research has examined expressions of anger towards computers, showing verbal and physical aggression towards computer equipment to be common (Charlton, 2009; Charlton et al., 2015). Withdrawal and regression are learned reactions associated with frustration where the individual anticipates failure or punishment in the face of barriers to their current goal (Britt and Janus, 1940; Lazar et al., 2006b). In turn the individual will act to move away from the current event that elicits frustration rather than tackling it (Britt and Janus, 1940). Such a response distances the individual from the event or environment that has lead to frustration, therefore returning the current ‘state of tension’ back to a previous equilibrium (Britt and Janus, 1940). In the context of digital technology this response has some precedence, with 10 per cent of American adults reporting that they did not go online, partially due to a frustrating experiences or a sense of overwhelming (Zickuhr, 2013). Fixation is seen as the maladaptive repetition of behaviour that has proved, at one time or another, to be successful in achieving goals. Individuals are often seen to attach themselves to responses that are wholly ineffective due to a pattern of intermittent reinforcement (some successful goal attainment in the past) or deficiencies in knowledge/skill set which are needed to respond to the new situation (Shorkey and Crocker, 1981). Shorkey and Crocker (1981) noted that individuals who experienced repeated frustrations exhibit deficiencies in effective goal-directed behaviour alongside losing all motivation to perform any type of this behaviour. This process ultimately results in resignation, and is indicative of a complete loss of motivation and hope, and according to Shorkey and Crocker, ‘non-involvement’.

Maladaptive responses to frustration can also be classified as being subjective in nature. The subjective, impunitive response is one where the individual experiences an aspect of embarrassment or shame as a result of the frustrating event. The individual may attempt to respond by passing frustration off as lightly as possible by making reference to ‘unavoidable circumstances’ e.g. “it couldn't be helped” (Shorkey and Crocker, 1981). The extrapunitive response shares some aspects with the objective aggression detailed above, and is an overt display of anger or condemnation directed towards the outside world e.g. persons, objects, and circumstances. In this case there is an attitude of hostility towards the environment e.g. “it's all your fault” or “I will get my own back”. Finally, the Intropunitive reaction is one that is littered with guilt and remorse, and is usually accompanied by a tendency for the individual to blame himself or herself as being at fault. ‘How could I have done a thing like that’, ‘I can never forgive myself’ (Shorkey and Crocker, 1981).

### 1.2 Responses to failures in digital technology

In the context of the present study, digital technology is defined as any device that functions using a binary computational code (including smartphones, laptops, computers), as well as services associated with such (e.g. the Internet, Wi-Fi, Social Networking). The research literature exploring frustration responses to failures in digital technology is very limited, but does present a basic foundation for exploring this area further. Frustrations with technology can be the result of factors internal to the end-user (poor training, lack of knowledge, and reticence to read relevant instructions) or external (flaws in computer hardware and software, failures in network integration, and malicious interventions such as malware) (Ceaparu et al., 2004). Other factors related to individual differences, such as self-efficacy, computer anxiety, and goal commitment (Lazar et al., 2006a,b; Ceaparu et al., 2004) have also been shown to influence the potential for the end user to experience frustrations.

Jokinen (2015) presented the term ‘frustration tendency’ in the context of the user experience. The term relates to the capacity for an individual to cope with the frustrating response, in turn reducing their level of frustration when they experience goal-incongruent events. Those individuals who have a higher frustration tendency are predicted to experience higher levels of frustration, presumed to be unable to cope with the emotions created as a result of the frustrating experience. In contrast, those who have a higher degree of competence, and who have a lower frustration tendency are better able to manage their emotions and cope with experiences that create frustration. However, Jokinen's work did not explore how individual differences could be used to predict such frustration tendencies, something that the current study aims to pursue further.

Paasonen (2015) explored the qualitative responses of individuals discussing their responses to technological failure. The work suggested that the emotive and visceral responses attached to technology failure are related to elements of uncertainty and instability in terms of the user's control. Many of the respondents used a variety of terms to explore aspects of failures with digital technology, including ‘dismay, horror, pain, distress, infuriation, fury, and helplessness’ (Paasonen, 2015, p. 705). Users also expressed an expectation that technology should just work seamlessly, with participants suggesting that failures with digital technology are something they come to expect. A dependency on digital technology also appeared to be related to failures, with many individuals discussing the notion of being cut off from the work and social relationships (Paasonen, 2015).

The experience of social isolation has links to the concept of Fear of Missing Out (FoMO). FoMO is often characterised by a desire to stay continually connected with what others are doing, particularly through the medium of social networking sites (SNSs) (Beyens et al., 2016; Przybylski et al., 2013). The notion of FoMO is typified by an individual's drive to constantly be connected with what others are doing in an online setting. Research has noted that those individuals who experienced higher levels of FoMO are consistently more likely to engage in problematic use of social media, including checking Facebook straight after waking up, before going to sleep and also during meals (Przybylski et al., 2013). High FoMO individual also reported being more likely to checking text messages, compose messages and emails whilst operating motor vehicles (Przybylski et al., 2013). These behavioural correlates suggest a potential for an individual who scores highly on a measure of FoMO to be more distracted in order to stay online and remain connected, but also more likely to take risks in order to achieve this goal. Individuals who experience higher levels of FoMO could have the potential to have more extreme reactions to failures with digital technology, particularly when such failures prevent them from accessing social media. This would also fit with the aspects of isolation and distress some participants talked about in Paasonen (2015).

An associated theme presented by Paasonen (2015) was that of dependency, where participants talked about a compulsion to use their digital devices and the anxiety that arose from not being able to use them, or access the services they wished due to a failure (Paasonen, 2015). Both dependency and compulsion have been associated with research on Internet addiction (e.g. Griffiths, 1996; Widyanto and Griffiths, 2006). The compulsion to engage in a particular activity fits into the aspect of salience proposed as one of the key criteria for assessing technology addiction. Here the activity which is the focus on the addiction becomes the most important activity for the individual, dominating thoughts, emotions, and behaviours. Symptoms of withdrawal are also seen as an aspect of addiction to technology, where removing the activity that is the focus of the addiction results in unpleasant feelings, including mood swings, irritability, and anxiety (Griffiths, 1996). Although the work by Paasonen (2015) provides an initial point from which to explore responses to failures in digital technology, it lacked direct and objective measures to explore the concepts of FoMO and Internet addiction further.

### 1.3 Personality factors and frustrations

The research exploring the connection to personality traits and frustration is sparse, and is limited to original work surrounding the nature of such traits. For example, McCrae and Costa (1987) suggested that those individuals who had higher levels of neuroticism had the tendency to adopt ‘inappropriate coping mechanisms’ and would be more likely to exhibit hostile reactions rather than deal with a disruptive emotion. They also go on to note that individuals exhibiting higher levels of neuroticism are more likely to adopt irrational beliefs or self-blame. Hence, from this perspective, it could be suggested that those individuals who score higher on levels of neuroticism are more likely to respond negatively to a failure with digital technology, either in terms of an aggressive response or one that is intropunitive in nature. Rose et al. (2002) noted that neuroticism was positively correlated with levels of frustration on perceived workload, but there was no direct link between the frustrating response and neuroticism. Graziano et al. (1996) noted that agreeable individuals are more likely to be able to control anger and be able to resolve situations that involve aspects of frustration, which may mean that such individuals are less likely to respond negatively to a frustration-eliciting event. Indeed, it has been further suggested that adult differences associated with the personality trait of agreeableness may indeed reflect a capacity to internally regulate aspects of anger and frustration (Graziano et al., 1996). The other personality factors that make up the Big-5 (e.g. openness, extraversion, and conscientiousness) have not been previously explored in the context of frustration responses, presenting these variables as potential elements for further investigation.

### 1.4 Aims and objectives

The aim of the current study was to explore if individual differences are related to the frustrating responses associated with failures in digital technology in everyday life. Jokinen (2015) highlighted that understanding individual differences in the context of the user experience are important, particularly the emotive aspects of such. The study proposed to create and pilot a new scale that measured the level of frustration an individual experiences as a result of failures with digital technology. In order to explore individual factors associated with responses to failures with digital technology, a variety of additional variables will be examined. Based on the previous work presented by Paasonen (2015), a formal test of the relationship between FoMO and Internet addiction will be conducted, and it is hypothesised that FoMO and Internet addiction will be significant predictors for maladaptive responses to failures in digital technology. Personality factors will also be explored, and will focus on the Big Five Personality traits presented by McCrae and Costa (1987). It is hypothesised, based on the original research from McCrae and Costa (1987), that higher levels of neuroticism will be associated with more extreme, maladaptive reactions to failures in digital technology. In contrast, higher levels of agreeableness will be associated with more adaptive reactions to failures in digital technology.

## 2 Method

### 2.1 Participants

In total 630 participants took part in an online survey between 24^th^ – 26^th^ April 2018 and were recruited through Qualtrics Participant Panels (https://www.qualtrics.com/uk/); participants were paid a small honorarium (£3.33) for their participation. Participants were aged between 18–68 years of age (Mean = 41.41, Std Dev. = 14.18), and were all resident in the UK. The survey took participants on average 16.28 minutes to complete. There was an equal distribution of males and females in the sample. All participants self-reported at least a basic level of expertise with digital technology. The Health and Life Sciences Ethical Committee at De Montfort University granted ethical approval for the current study.

### 2.2 Materials

#### 2.2.1 Response to failures in digital technology scale (RFDT)

For the purposes of the current study, a scale was designed that aimed to examine self-reported responses to failures with digital technology. In order to provide a theoretical grounding for the scale, the original framework categorising responses to frustration was used (Britt and Janus, 1940; Shorkey and Crocker, 1981). Items were constructed around two key categories of maladaptive and adaptive responses. Maladaptive responses included those behaviours that often lead to making the situation worse, often by creating additional problems. Items associated with this category included ‘get angry’, ‘panic’ or ‘feel depressed’, with seventeen items exploring these responses. In contrast, adaptive responses are typified by an attempt on the part of the individual to solve the problem or circumvent the issue, with nine items measuring these responses.

Participants were introduced to the scale and asked to think about their experiences associated with failures in digital technology. A definition of what digital technology referred to was included in this introductory section, along side relevant examples of a failure (e.g. software or an application not responding or crashing, poor Wi-Fi or Internet access and issues with hardware, such as poor battery life). Respondents were asked to recall more general experiences related to failures in digital technology rather than being asked to respond to specific types of failure in this instance. Responses were scored on a 5-point Likert Scale (1 = Very Unlikely, 5 = Very likely), with responses being classified as adaptive being reversed scored (see Supplementary Material for the full scale). A final 26-item scale was produced, with a high score was indicative of a more extreme, maladaptive response to failures in digital technology, with scores ranging from 26 to 130.

#### 2.2.2 Fear of Missing Out scale (FoMOs)

Fear of missing out was assessed using the 10-item FoMOs by Przybylski et al. (2013). Participants are asked to respond to statements related to missing out on important information and fears relating to friends having more rewarding experiences than themselves. The original study reported good internal reliability for the scale (= .90)**,** with the present study reporting an internal reliability of α = .91. Scores on this scale were used as a continuous variable in the correlation and regression analyses, with higher scores indicating higher FoMO.

#### 2.2.3 Online cognition scale (OCS)

Davis et al. (2002) presented the 36-item OCS as a mechanism that explores aspects of problematic Internet use. In the study by Davis et al. (2002) the OCS demonstrated a high level of internal consistency as a measure for problematic Internet use with a Cronbach's alpha of 0.94, with the present study reporting a Cronbach's Alpha of 0.97. Possible scores on the OCS range between 36 to 252.

#### 2.2.4 Big 5 personality inventory (BFI)

The BFI, a 44-item inventory that measures an individual according five key dimensions of personality (John and Srivastava, 1999), was used. Items are scored on a five point Likert-scale (from 1 = Strongly Disagree, to 5 = Strongly Agree; see John and Srivastava, 1999 for additional scoring information). The inventory presents total scores for extraversion, agreeableness, neuroticism, openness, and conscientiousness. The stability of such personality traits in an adult population has been previously demonstrated (Cobb-Clark and Schurer, 2012). In the context of the present study the BFI was used to explore the structural relationships between individual personality constructs and online deception, and not as a conclusive measure of personality.

## 3 Results

The aim of this study was to explore end user responses to failures in digital technology and the relationship with personality, Internet addiction, and Fear of Missing out. Descriptive statistics and Pearson's correlations for the key variables are shown in Table 1, where *n* = 630. Strong positive correlations were noted between FoMO, OCS, Neuroticism and total scores on the RFDT scale. This indicates that as individuals score higher on aspects of FoMO, Internet addiction and neuroticism, they are more likely to exhibit more extreme, negative, and maladaptive responses to failures in digital technology. In contrast, negative correlations were noted between agreeableness, conscientiousness, and total scores on the RFDT, suggesting those individuals scoring higher in these traits are more likely to have less extreme, adaptive responses to failures in digital technology. Age was also negatively correlate with more extreme responses to failures in digital technology, showing that as age increases, the level of frustration that an individual experiences decreases.

**Table 1:** Descriptive statistics and correlations.

Measure	1	2	3	4	5	6	7	8	9
1. Age	-								
2. FoMO	−.365**	-							
3. Frust	−-.234**	.546**	-						
4. OCS	−.341**	.628**	.469**	-					
5. Extraversion	0.29	.070	−.064	.017	-				
6. Agreeableness	.231**	−.261**	−.371**	−.239**	.221**	-			
7. Conscientiousness	.321**	−.333**	−.459**	−.286	.307**	.525**	-		
8. Neuroticism	−.227**	.294**	.456**	−.275	−.455**	−.458**	−.458**	-	
9. Openness	−.035	.045	−.053	.135**	.372**	.206**	.206**	−.095*	-
**Mean**	**41.41**	**24.78**	**61.88**	**127.36**	**23.96**	**32.30**	**32.34**	**23.28**	**32.82**
**SD**	**14.18**	**9.07**	**15.06**	**45.38**	**5.84**	**6.14**	**5.95**	**6.00**	**6.12**

* = p < .05; **p < .001.

The mean score on the RFDT for males was 61.38 (SD = 16.06), and for females it was 62.38 (SD = 13.99). Sex differences related to the level of frustration experienced as a result of a failure in digital technology were further explored using an independent samples t-test. This indicated that there were no significant differences between males and females according the level of frustration they experienced as a result of failures in digital technology (*t* (628) = −.838, p > .05). As there were no significant differences for sex, this variable was excluded from further analyses.

### 3.1 Internal reliability and factor analysis for the responses to failures with digital technology (RFDT) scale

The 26-item RFDT was developed to explore self-reported responses to failures with digital technology. In the context of the current study, the scale had good internal reliability, with a Cronbach's α of .80. The Kaiser-Myer-Oklin value obtained for the scale items was .913, exceeding the recommended value of .60 (Kaiser, 1970). Furthermore, Bartlett's Test of Sphericity was also found to be significant (χ^2^(325) = 8425.312, *p* < .001) suggesting that the scale was suitable for factor analysis.

The scale was subjected to Principal Components Analysis using a direct oblimin rotation. The analysis revealed four key factors that accounted for a total of 58.60% of the observed variance. The factor loadings for the scale are presented in Table 2. The first factor, accounting for 31.82% of the variance is labelled as ‘Maladaptive responses’ to failures with digital technology. This factor is dominated by emotive responses to the failure, including aspects associated with withdrawal, regression, and fixation (Britt and Janus, 1940). A second factor that accounted for 14.50% of the variance was labelled as ‘Adaptive’ responses, an include a variety of active attempts find a solution to the failure, including using a variety of online resources to search for help. A third factor, accounting for 6.80 % of the observed variance, was labelled as ‘Externalising support and venting frustrations’. On the one hand this factor contained items that were associated with extrapunitive reactions, focused on voicing frustrations on social media or targeting an individual believed to be responsible for the failure. Two further items related to reaching out to external support, either via social media or through paying someone to fix the issue. The fourth factor, accounting for 4.80% of the observed variance was labelled as Anger and Resignation – in this factor aspects of anger, annoyance and resignation were all featured.

**Table 2:** Factor loadings for exploratory factor analysis with oblimin rotation of responses to failures with digital technology scale.

	Maladaptive responses	Adaptive Responses	External Support and Venting	Anger and Resignation
Feel that it is my fault	**.844**	−.064	.018	−.099
Feel lonely	**.824**	.040	−.104	−.168
Feel depressed	**.777**	.097	−.014	.089
Worry that I have done something wrong	**.763**	−.126	.065	−.027
Begin to feel that I am useless	**.758**	.136	−.136	−.135
Become withdrawn	**.704**	.089	−.109	.071
Panic	**.664**	.015	−.011	.193
Obsess about the issue	**.619**	−.175	.154	.201
Feel like I am missing out on something	**.569**	−.106	−.127	.110
Become annoyed with myself	**.504**	−.115	.010	.349
Lose focus on the task I should be doing	**.467**	−.075	.041	.418
Throw objects or damage things	**.423**	.208	−.360	.134
Try everything I can to fix the issue∗	.011	**.790**	−.181	−.178
Go online to find a solution∗	.104	**.787**	−.032	−.096
Try to search for a solution to the problem∗	.006	**.781**	−.252	−.169
Use an online help forum to see if I can find a solution∗	.110	**.748**	.180	−.040
Use the situation as a learning experience in order to expand my knowledge∗	−.043	**.714**	.163	.137
Look for someone who has experienced a similar problem to see how they solved it∗	−.089	**.678**	.193	.032
Relish the opportunity to solve the issue∗	−.157	**.552**	.080	.177
Post on social media to make my frustrations about the problem clear	.097	−.002	−**.815**	.022
Post on social media to see if someone else can help me∗	−.033	.191	**.794**	.102
Make my unhappiness clear to the person or company I feel is responsible	−.035	−.035	−**.642**	.400
Try to pay someone to fix the issue∗	−.260	.096	**.442**	−.079
Get angry	.098	−.052	.076	**.764**
Feel annoyed as I am paying for something that should work	−.013	−106	−.216	**.750**
Give up and go and do something else	.249	.126	−.135	**.431**

Note: Factor loadings >.40 are in boldface.

∗ Indicates a reverse scored item.

### 3.2 Individual differences and FRDT

In order to examine how the key predictors variables for this study impacted on self-reported responses to failures with digital technology, a simultaneous multiple regression was conducted. In the absence of any clear theoretical literature in this area, all variables were entered in one stage. The Durbin-Watson statistic was 1.928, suggesting that independence of errors could be assumed, and values of tolerance and VIF suggested that multicollinearity was not a concern (VIF average = 1.55, tolerance average = 0.66).

The results of the regression are displayed in Table 3. With all of the key predictor variables included, the overall the model explained 42% of the variance in total scores on the RFDT scale. FoMO, OCS, extraversion, and neuroticism all acted as significant positive predictors for scores on the RFDT. In contrast, agreeableness, conscientiousness, and openness all acted as significant negative predictors for scores on the RFDT. With all other variables entered into the regression, age acted as a significant predictor for scores on the RFDT, showing that as age increased, individual were more likely to exhibit maladaptive responses to failures with digital technology.

**Table 3:** Summary of hierarchical regression for variables predicting total RFDT (n = 630).

Variable	β	*t*
**Step 1**	*F* (_7*, 622*_) = 64.844, *R*^2^ = .422***	
Age	.071	2.09*
FoMO	.256	6.15***
OCS	.108	2.67**
Extraversion	.137	3.63***
Agreeableness	−.147	−4.01***
Conscientiousness	−.168	−4.17***
Neuroticism	.307	7.99***
Openness	−.092	−2.71**

*p < 0.05 **p < 0.01, ***p < 0.001.

## 4 Discussion

The current study explored frustrations associated failures in digital technology could be related to individual differences in personality traits, fear of missing out, and Internet addiction. The study also presents the first stage in the development of a scale that aims to measure subjective responses to failures in digital technology. Each of these aims and objectives will be discussed in turn alongside the results for the current study.

### 4.1 Frustrations, FoMO and Internet addiction

The finding that both FoMO and Internet addiction are both significantly correctly with more extreme, maladaptive responses to failures in digital technology shares some links to previous work by Paasonen (2015). In Passonen's work, participants presented essay-based explorations of how failures or malfunctions in aspects related to digital technology served to make them feel. Those individuals who spend a great deal of time engaged in activities that have digital technology as a critical component, particularly in terms of social networking or as a connection to the outside world, are more likely exhibit a greater dependency on such technology. Inhibiting access to such systems as a result of failure for those who exhibit aspects of FoMO or Internet addiction presents could cause severe anxiety and symptoms of withdrawal (Kuss et al., 2014). The finding is another step in advancing our understanding of the relationships between FoMO, Internet addiction and dependency on digital technology. It could also be useful as a framework for developing techniques and strategies that may limit more extreme, maladaptive responses to failures in digital technology. Both fear of missing out and Internet addiction are driven by a need to stay online and stay connected at any cost. In the instance of FoMO, individuals begin to experience anxiety when their capacity to engage with their online social environment is diminished or removed. Many of the maladaptive responses highlighted in the RFDT scale are visceral and emotive in nature, closely aligned to an individual who is experiencing a high degree of anxiety as a response to technology failure. Both Internet addiction and FoMO have a compulsion to engage with aspects of digital technology in order to stay online, in the latter case in order to stay connect with social activities other may be having. Removing such access, or the facility to engage with online activities that fulfil the addiction could result in the manifestation of withdrawal, alongside aspects of anxiety. It is therefore reasonable to expect aspects of both FoMO and Internet addiction to be key predictors for maladaptive responses to failures associated in aspects of digital technology that service an individual's need to stay online.

### 4.2 Personality and response to failures in digital technology

In the context of personality factors, the results from the present study do support some previous research that has explored how such traits govern responses to frustration. The finding that neuroticism acted as a positive predictor for maladaptive responses to failures in digital technology aligns well with the original conceptualisation of this personality trait (McCrae and Costa, 1987). In their view, an individual who scores higher on neuroticism was more frequently seen to use inappropriate coping mechanisms and hostile reactions to aspects associated with frustration. These are very clearly the reactions that have been isolated on the maladaptive component of the RFDT scale. They further noted that higher levels of neuroticism are linked to irrational beliefs, including a tendency to self-blame, an aspect that also fits into the maladaptive, subjective category, particularly that of the intropunitive response. Interestingly only two other personality factors acted as significant negative predictors for scores on the RFDT scale, these being agreeableness and conscientiousness. Agreeableness has been previously associated with the capacity for an individual to inhibit or internalise aspects of anger and negative affect when a situation produces frustration (Graziano et al., 1996). Those individuals who are highly agreeable are potentially less likely to engage in overt displays of maladaptive responses to failures with digital technology, an aspect that is supported by the findings from the current research. Conscientiousness has been previously associated with the agreeableness, so the finding that both are key predictors for responses to failures in digital technology is a logical one. From these findings, it would appear that those individuals better able to deal effectively with the frustrating response, and who can deal with such in a more constructive manner are less likely to respond in a maladaptive way.

### 4.3 Responses to failures with digital technology scale

As a first attempt to quantify how individuals respond to failures associated with forms of digital technology, the scale used in the current study provides a potential step in exploring the phenomena further. The scale presents a number of factors that share links to previous research exploring the responses to frustration. There is clearly a distinction between the factors in the present scale, with most of the items loading directly onto maladaptive, emotive response to frustration. These items cover a variety of maladaptive, objective responses originally identified by Britt and Janus (1940), as well as some items that related directly to intropunitive responses (e.g. ‘I feel that it is my fault’). It appears that, in the context of the current scale, the distinction between maladaptive objective and subjective responses to frustration are not as clear-cut as the distinction made by Britt and Janus (1940). A second factor included items that clearly capture the element of an adaptive response. In the original framework, adaptive responses are seen as the potential to transform the ‘energy’ resulting from the frustrating response into a more productive mechanism that aids goal attainment or circumvention of the frustration-eliciting event (Britt and Janus, 1940; Shorkey and Crocker, 1981). These aspects are adequately captured in the items that load onto this factor, including a search for a potential solution online, or relishing the chance to engage in problem solving to resolve the issue. The third factor appears to be typified by attempt to vent frustrations to an external agent, either directly to the company or via social media. Two additional items detail the use of social media to help find a potential solution to the problem, or an attempt to overcome the issue by paying someone to help. Research has previously shown that consumers will often turn to social media in an attempt to both vent their frustrations, particularly when they feel like they have been ignored by an organisation (Tripp and Grégoire, 2011). Such responses can have a particularly damaging impact on brand reputation, especially where the reply to such public venting are not carefully managed (Grégoire et al., 2015). The final factor appears to be a mix of anger and annoyance, but also an aspect of resignation that the issue might not get solved, hence it is better to go and do something else. The anger response to frustrations with digital technology is something that has been previously explored, with researchers noting that some individual adopt a perception that computers have human like characteristics (Nass and Moon, 2000). This phenomenon has been termed ethopoeia, and research has noted that anger intensity related to frustrating experiences can be influenced by the extent to which an individual believes the computer is responsible for such (Charlton, 2009; Charlton et al., 2015).

### 4.4 Limitations

The RFDT scale in its current form presents an initial start point for further exploring self-reported responses to failures with digital technology. However, in light of the factor analysis for the scale, further development of the scale is essential. Primarily, the scale itself has a clear imbalance between aspects of maladaptive and adaptive frustration responses. This could of course, in turn, presented a scale that is more inclined to reflect more maladaptive responses to failures in digital technology. Aligned with this limitation, there may also be worth in adding extra items that could potentially load onto the two weaker factors included in the scale.

The use of self-report also presents another possible limitation to the current study, particularly when individuals may wish to make their feelings about a particularly frustrating experience known. In the absence of a viable alternative, such as diary-based records for exploring how individuals actually reacted to specific failures with digital technology, the use of self-report data was considered the most appropriate approach for this study. However, further research in this area could seek to adopt a more formal way of recording incidences of failures in digital technology and the associated responses to such.

## 5 Conclusion

The present research presents a very useful picture of how individual differences serve to shape end user reactions to failures with digital technology. From a practical perspective, it is clear that maladaptive responses to failures associated with digital technology can have a detrimental impact on productivity and goal attainment (Zimmerman et al., 2014). In the in the context of an organisation this could in turn lead to withdrawal and poor job performance. Similarly, these findings can be integrated into work already carried out exploring emotions in the context of user experiences. For example, the work by Jokinen (2015) in relation to the competence-frustration model highlights the influence negative affect can have on an individual's experience with a system. Negative experiences can serve to reduce confidence in using technology, and create a poorer user experience. Understanding factors that may lead some individuals to experience more frustration compared to others could therefore have clear practical implications. Finding a way to bolster more productive, adaptive responses to failures with digital technology presents a potential to mitigate such losses in production, as well as enhancing organisational resilience. The potential for service disruption and failures to be caused by malicious interventions (e.g. malware, Denial of Service attacks, Cyberterrorism) has also been raised as a key concern (HM Government, 2016). By developing an understanding of how individuals respond to such attacks can also serve to bolster national responses to these threats, ensuring that infrastructure remains protected and to ensure the potential to make these issues even worse is avoided.

## Declarations

### Author contribution statement

Lee Hadlington, Mark O. Scase: Conceived and designed the experiments; Performed the experiments; Analyzed and interpreted the data; Contributed reagents, materials, analysis tools or data; Wrote the paper.

### Funding statement

This research did not receive any specific grant from funding agencies in the public, commercial, or not-for-profit sectors.

### Competing interest statement

The authors declare no conflict of interest.

### Additional information

No additional information is available for this paper.

## References

[bib1] Berkowitz L. (1989). Frustration-aggression hypothesis: examination and reformulation. Psychol. Bull..

[bib2] Beyens I., Frison E., Eggermont S. (2016). “I don’t want to miss a thing”: adolescents’ fear of missing out and its relationship to adolescents’ social needs, Facebook use, and Facebook related stress. Comput. Hum. Behav..

[bib3] Bonne O., Grillon C., Vythilingam M., Neumeister A., Charney D.S. (2004). Adaptive and maladaptive psychobiological responses to severe psychological stress: implications for the discovery of novel pharmacotherapy. Neurosci. Biobehav. Rev..

[bib4] Borsook D., Maleki N., Becerra L., McEwen B. (2012). Understanding migraine through the lens of maladaptive stress responses: a model disease of allostatic load. Neuron.

[bib5] Britt S.H., Janus S.Q. (1940). Criteria of frustration. Psychol. Rev..

[bib6] Buck R., Khan M., Fagan M., Coman E., Buck R. (2017). The user affective experience Scale: a measure of emotions anticipated in response to pop-up computer warnings the user affective experience Scale: a measure of emotions anticipated in response to pop-up computer warnings. Int. J. Hum. Comput. Interact..

[bib7] Ceaparu I., Lazar J., Bessiere K., Robinson J., Shneiderman B. (2004). Determining causes and severity of end-user frustration. Int. J. Hum. Comput. Interact..

[bib8] Charlton J.P. (2009). The determinants and expression of computer-related anger. Comput. Hum. Behav..

[bib9] Charlton J.P., Kappas A., Swiderska A. (2015). Does computing anger have social elements? A comparison with driving anger. Behav. Inf. Technol..

[bib10] Cobb-Clark D.A., Schurer S. (2012). The stability of big-five personality traits. Econ. Lett..

[bib11] Graziano W.G., Jensen-Campbell L.A., Hair E.C. (1996). Perceiving interpersonal conflict and reacting to it: the case for agreeableness. J. Pers. Soc. Psychol..

[bib12] Grégoire Y., Salle A., Tripp T.M. (2015). Managing social media crises with your customers: the good, the bad, and the ugly. Bus. Horiz..

[bib13] Griffiths M. (1996). Behavioural addiction: an issue for everybody?. J. Workplace Learn..

[bib14] Gross M.L., Canetti D., Vashdi D.R. (2016). The psychological effects of cyber terrorism. Bull. At. Sci..

[bib15] Gross M.L., Canetti D., Vashdi D.R. (2017). Cyberterrorism: its effects on psychological well-being, public confidence and political attitudes. J. Cybersecur..

[bib16] HM Government (2016). National Cyber Security Strategy. https://www.gov.uk/government/uploads/system/uploads/attachment_data/file/567242/national_cyber_security_strategy_2016.pdf.

[bib17] John O.P., Srivastava S. (1999). Big five inventory (Bfi). Handb. Pers. Theory Res..

[bib18] Jokinen J.P.P. (2015). Emotional user experience: traits, events, and states. Int. J. Hum. Comput. Stud..

[bib19] Kaiser H.F. (1970). A second-generation little jiffy. Psychometrika.

[bib20] Kuss D.J., Griffiths M.D., Karila L., Billieux J. (2014). Internet addiction: a systematic review of epidemiological research for the last decade. Curr. Pharmaceut. Des..

[bib21] Lazar J., Jones A., Hackley M., Shneiderman B. (2006). Severity and impact of computer user frustration: a comparison of student and workplace users. Interact. Comput..

[bib22] Lazar J., Jones A., Shneiderman B. (2006). Workplace user frustration with computers: an exploratory investigation of the causes and severity. Behav. Inf. Technol..

[bib23] McCrae R.R., Costa P.T. (1987). Validation of the five-factor model of personality across instruments and observers. J. Pers. Soc. Psychol..

[bib24] Nass C., Moon Y. (2000). Machines and mildlessness: social responses to computers. J. Soc. Issues.

[bib25] Paasonen S. (2015). As networks fail: affect, technology, and the notion of the user. Televis. New Media.

[bib26] Przybylski A.K., Murayama K., Dehaan C.R., Gladwell V. (2013). Motivational, emotional, and behavioral correlates of fear of missing out. Comput. Hum. Behav..

[bib27] Rose C.L., Murphy L.B., Byard L., Nikzad K. (2002). The role of the big five personality factors in vigilance performance and workload. Eur. J. Pers..

[bib28] Rosenzweig S. (1934). Types of reaction to frustration. J. Abnorm. Soc. Psychol..

[bib29] Saariluomaand P., Jokinen J.P.P. (2014). Emotional dimensions of user experience: a user psychological analysis. Int. J. Hum. Comput. Interact..

[bib30] Shorkey C.T., Crocker S.B. (1981). Frustration theory: a source of unifying concepts for generalist practice. Soc. Work.

[bib31] Tripp T.M., Grégoire Y. (2011). When unhappy customers strike back on the internet. MIT Sloan Manag. Rev..

[bib32] Widyanto L., Griffiths M. (2006). “Internet addiction”: a critical review. Int. J. Ment. Health Addiction.

[bib33] Zickuhr K. (2013). Who’s Not Online and Why.

[bib34] Zimmerman N.K., Sambrook E., Gore J.S. (2014). The effects of a computer malfunction on subsequent task performance. Behav. Inf. Technol..

