# Supplementary material for: End-user frustrations and failures in digital technology: exploring the role of Fear of Missing Out, Internet addiction and personality
Source: Heliyon. 2018 Nov 1;4(11):e00872. doi: 10.1016/j.heliyon.2018.e00872 (PMC6223105; doi:10.1016/j.heliyon.2018.e00872)
Supplement: Supplementary Material [file mmc1.docx]

**Supplementary Material: End-User Frustrations and failures in digital technology: Exploring the role of Fear of Missing out, Internet addiction and Personality.**

**Responses to Failures in Digital Technology Scale**

From time to time, modern digital technology doesn’t always work in the way we want it to, and fails to help us reach our goals and objectives. This questionnaire asks you about your experience of such failures with digital technology and more importantly how you respond to them.

For the current questionnaire we want you to focus directly on aspects of ***digital technology*** – this relates to computer-based products and services, including (but not limited to) smartphones, tablets, PCs, Apps and Software, the Internet and WiFi to give you a few examples.

We want you to think about how you respond to a ***failure with digital technology***. This could include:

Software or an application crashing or not responding

Applications being slow to load

Poor Wifi or Internet Access

Issues with hardware, such as poor battery life.

With these examples in mind and using the scale provided, rate how likely you are to do the following in response to a failure with digital technology.

When there is a problem or issue related to digital technology I:

|  | Very Unlikely (1) | Somewhat Unlikely (2) | Neither Likely nor Unlikely (3) | Somewhat Likely (4) | Very Likely (5) |
| --- | --- | --- | --- | --- | --- |
| 1. Become withdrawn |  |  |  |  |  |
| 2. Feel depressed |  |  |  |  |  |
| 3. Panic |  |  |  |  |  |
| 4. Feel lonely |  |  |  |  |  |
| 5. Feel like I am missing out on something |  |  |  |  |  |
| 6. Relish the opportunity to solve the issue ^ |  |  |  |  |  |
| 7. Become annoyed with myself |  |  |  |  |  |
| 8. Lose focus on the task I should be doing |  |  |  |  |  |
| 9. Give up and go and do something else |  |  |  |  |  |
| 10. Obsess about the issue |  |  |  |  |  |
| 11. Feel that it is my fault |  |  |  |  |  |
| 12. Worry that I have done something wrong |  |  |  |  |  |
| 13. Try to search for a solution to the problem ^ |  |  |  |  |  |
| 14. Try everything I can to fix the issue |  |  |  |  |  |
| 15. Throw objects or damage things |  |  |  |  |  |
| 16. Feel annoyed as I am paying for something that should work |  |  |  |  |  |
| 17. Try to pay someone to fix the issue ^ |  |  |  |  |  |
| 18. Go online to find a solution ^ |  |  |  |  |  |
| 19. Look for someone who has experienced a similar problem to see how they solved it ^ |  |  |  |  |  |
| 20. Use an online help forum to see if I can find a solution ^ |  |  |  |  |  |
| 21. Post on social media to see if someone else can help me ^ |  |  |  |  |  |
| 22. Post on social media to make my frustrations about the problem clear |  |  |  |  |  |
| 23. Make my unhappiness clear to the person or company I feel is responsible |  |  |  |  |  |
| 24. Begin to feel that I am useless |  |  |  |  |  |
| 25. Use the situation as a learning experience in order to expand my knowledge ^ |  |  |  |  |  |
| 26. Get angy |  |  |  |  |  |

^ = reversed scored item
